# Reliability of AI Tools in Generating Patient Education Brochures for Bariatric Surgery: An Observational Study

**DOI:** 10.7759/cureus.99321

**Published:** 2025-12-15

**Authors:** Sneha Zakkir, Khushali Dadhich, Bavanthi K V, Lakshmi Nikhita Bukkasamudram, Anjali Krishna Santhosh, Shrirampirajin Thangaraj, Pallavi Padmakar Kulkarni

**Affiliations:** 1 General Surgery, Church of South India Hospital, Bangalore, IND; 2 General Surgery, K.J. Somaiya Hospital and Research Centre, Mumbai, IND; 3 General Surgery, SRM Medical College Hospital and Research Centre, Chennai, IND; 4 General Surgery, International Higher School of Medicine, Bishkek, KGZ; 5 General Surgery, Kerala Institute of Medical Science, Trivandrum, IND; 6 General Surgery, Lifeline Multispecialty Hospital, Chennai, IND; 7 General Surgery, Plastic Surgery, Grant Medical College (GMC) Mumbai, Sir J.J. Hospital Mumbai, Mumbai, IND

**Keywords:** artificial intelligence (ai), bariatric & metabolic surgery, chatgpt, google gemini, patient education, patient education brochure

## Abstract

Background: Patient education plays a key role in helping individuals understand their health conditions and participate in treatment. With the growing use of artificial intelligence (AI), tools like ChatGPT (OpenAI, San Francisco, CA, USA) and Google Gemini (Mountain View, CA, USA) are increasingly being used to generate patient information. This study assessed how readable and reliable AI-generated brochures on bariatric surgery are.

Methods: A cross-sectional study was conducted in September 2024 to generate patient brochures on six common bariatric procedures using ChatGPT and Google Gemini. Each brochure was evaluated for readability using the Flesch-Kincaid metrics, and “similarity” was assessed using Quillbot to estimate text overlap with existing literature (higher similarity indicating greater overlap). Reliability was measured using the modified DISCERN score, where higher scores reflect more trustworthy health information.

Results: ChatGPT generally produced longer brochures with more sentences, while Gemini generated shorter text with slightly longer sentences. Despite these structural differences, both tools produced content with similar readability levels - approximately college-level - and comparable reliability scores. Gemini showed higher similarity with pre-existing text, while ChatGPT produced more original phrasing. Overall, both tools generated patient information of good reliability but with limited accessibility for individuals with lower literacy.

Conclusion: ChatGPT and Google Gemini can produce reliable educational material on bariatric surgery, but the readability remains higher than ideal for the average patient. Human editing and simplification may be necessary to make AI-generated brochures more accessible and suitable for routine patient education.

## Introduction

Patient education is an essential component of healthcare, enabling individuals to understand their health conditions, participate in treatment, and work toward better outcomes [1]. It supports comprehension of symptoms and diagnoses, promotes treatment adherence, encourages preventive care, improves communication with healthcare providers, and builds confidence in decision-making, ultimately reducing morbidity and mortality [2,3]. In the current age, patients are increasingly proactive and seek to remain informed throughout their care.

Artificial intelligence (AI) refers to the use of computer systems to simulate intelligent behavior [4]. It has enhanced automation, efficiency, and decision-making through machine learning and data analytics [5]. Among commonly used AI tools are ChatGPT (OpenAI, San Francisco, CA, USA) and Google Gemini (Mountain View, CA, USA), both of which use natural language processing and deep learning to generate human-like responses [6,7]. While AI contributes to various clinical and administrative functions in healthcare - from robotic-assisted procedures to electronic medical records [4] - its growing relevance lies in its ability to instantly provide patients with explanations about medical conditions, diagnosis, and prognosis [8,9]. This makes AI an increasingly influential tool in patient communication and education.

Bariatric surgery, also known as metabolic or weight-loss surgery, includes several procedures that help manage morbid obesity [10]. It is effective for weight reduction, improving metabolic diseases, and reducing long-term mortality [11-13]. It also decreases the risk of comorbidities such as obstructive sleep apnea, diabetes, cardiovascular disease, hypertension, and gastroesophageal reflux disease [14]. Despite these benefits, bariatric surgery remains underutilized [15], partly due to limited patient awareness and understanding - highlighting the need for accessible educational resources, including those generated using AI.

Although AI offers rapid access to health information, concerns persist regarding patient confidentiality, variable accuracy, contextual limitations, and inconsistent responses [16]. Evaluating the quality of AI-generated educational materials is therefore necessary.

This study compared the readability, reliability, and accuracy of bariatric surgery patient education brochures generated by ChatGPT and Google Gemini.

## Materials and methods

Study design and setting

This was a cross-sectional original research study conducted over one week beginning on 29th September 2024. As the study involved only AI-generated text and did not include human participants, Ethics Committee approval was not required [17,18].

Data sources and AI tools used

Data were obtained from two large language models: ChatGPT version 4.0 and Google Gemini 1.5 Flash. These tools were selected to compare two widely used AI platforms capable of generating patient education materials.

Selection of bariatric procedures

Six bariatric procedures - gastric bypass surgery, sleeve gastrectomy, endoscopic sleeve gastrectomy, adjustable gastric banding, biliopancreatic diversion with duodenal switch, and intragastric balloon - were chosen because they represent the most commonly performed and clinically relevant surgeries in bariatric practice. The findings were intended to reflect procedures that patients frequently encounter in clinical settings.

Standardization of prompts

To minimize bias, all prompts were standardized and phrased identically across both AI tools. Each prompt was entered once without rephrasing, follow-up questions, or clarifications. The following prompts were used: “Write a patient education guide for Gastric Bypass Surgery”, “Write a patient education guide for Sleeve Gastrectomy”, “Write a patient education guide for Endoscopic Sleeve Gastrectomy”, “Write a patient education guide for Adjustable Gastric Banding (Lap Band)”, “Write a patient education guide for Biliopancreatic Diversion with Duodenal Switch (BPD/DS)” and “Write a patient education guide for Intragastric balloon.” All responses were saved in Microsoft Word.

Variables and measurements

The brochures were assessed for structural and readability parameters using the Flesch-Kincaid Calculator [19], which provided word count, sentence count, average words per sentence, syllables per word, ease of understanding, and overall grade level. Similarity percentage was calculated using the Quillbot Plagiarism tool, where higher values indicate greater overlap with existing online text and lower percentages reflect more original phrasing [20]. Reliability was assessed using the modified DISCERN score, a validated 5-point scale evaluating the trustworthiness of health information [21]. Each of the five parameters is scored “yes” (1 point) or “no” (0 points), with total scores ranging from 0 (poor reliability) to 5 (high reliability).

Data management

All extracted data were transferred into Microsoft Excel (Redmond, WA, USA) prior to analysis.

Statistical analysis

Statistical analysis was conducted using R version 4.3.2 (R Foundation for Statistical Computing, Vienna, Austria). An independent (unpaired) t-test was used to compare mean values between ChatGPT and Google Gemini. Normality and variance assumptions were checked prior to testing. A p-value <0.05 was considered statistically significant. Pearson’s coefficient was used to assess correlation between readability (ease score) and reliability (mDISCERN score).

Bias and quality control

Standardizing prompts, avoiding follow-up interactions, and analyzing identical surgical topics across both AI tools helped reduce measurement bias. Automated tools (Flesch-Kincaid, Quillbot, and mDISCERN) ensured consistent evaluation of all outputs.

## Results

A total of six patient education brochures were generated for common bariatric procedures - gastric bypass surgery, sleeve gastrectomy, endoscopic sleeve gastrectomy, adjustable gastric banding, biliopancreatic diversion with duodenal switch (BPD/DS), and intragastric balloon - using ChatGPT and Google Gemini. Each response was analyzed for structural characteristics, readability, similarity, and reliability.

Descriptive characteristics of text output

ChatGPT generated brochures with significantly higher word and sentence counts compared to Gemini. The mean word count for ChatGPT was 570.17 ± 57.53, whereas Gemini produced 412.50 ± 59.66 words (p = 0.001). Similarly, the number of sentences was greater for ChatGPT (59.67 ± 13.32) than for Gemini (35.00 ± 5.97; p = 0.002). In contrast, Gemini demonstrated longer sentence construction, with a greater average words per sentence (11.88 ± 1.06 vs. 9.82 ± 1.73; p = 0.032). The mean syllables per word were comparable between the two tools (p = 0.283). These findings are summarized in Table 1.

**Table 1:** Characteristics of responses generated by ChatGPT and Google Gemini * Independent-sample t-test; p < 0.05 considered statistically significant.

Parameter	ChatGPT		Gemini		t-value	p-value *
	Mean	SD	Mean	SD		
Words	570.17	57.53	412.5	59.66	5.48	0.001*
Sentences	59.67	13.32	35	5.97	4.93	0.002*
Average Words per Sentence	9.82	1.73	11.88	1.06	2.63	0.032*
Average Syllables per Word	1.97	0.14	1.88	0.12	1.12	0.283
Grade Level	11.45	1.32	11.28	1.44	0.21	0.839
Ease Score	30.48	10.62	35.45	9.95	0.83	0.423
Similarity %	17.52	9.92	34.43	17.84	2.17	0.07
Reliability Score	3.83	0.4	4	0	1.03	0.333

Readability and ease of comprehension

Both AI tools generated brochures with a Flesch-Kincaid grade level corresponding to college-level comprehension (ChatGPT: 11.45 ± 1.32; Gemini: 11.28 ± 1.44; p = 0.839). Ease of reading scores indicated slightly greater accessibility for Gemini (35.45 ± 9.95) compared to ChatGPT (30.48 ± 10.62), but the difference was not statistically significant (p = 0.423).

Similarity analysis

Gemini demonstrated a higher similarity percentage with pre-existing literature (34.43 ± 17.84%) than ChatGPT (17.52 ± 9.92%). This difference approached statistical significance (p = 0.07), suggesting ChatGPT produced more original phrasing while Gemini generated text with greater overlap.

Reliability assessment

The modified DISCERN score demonstrated good reliability for brochures from both tools, with ChatGPT scoring 3.83 ± 0.40 and Gemini achieving 4.00 ± 0.00 (p = 0.333). These results indicate that both AI tools consistently produced information of good quality and relevance for patient education.

**Figure 1:**
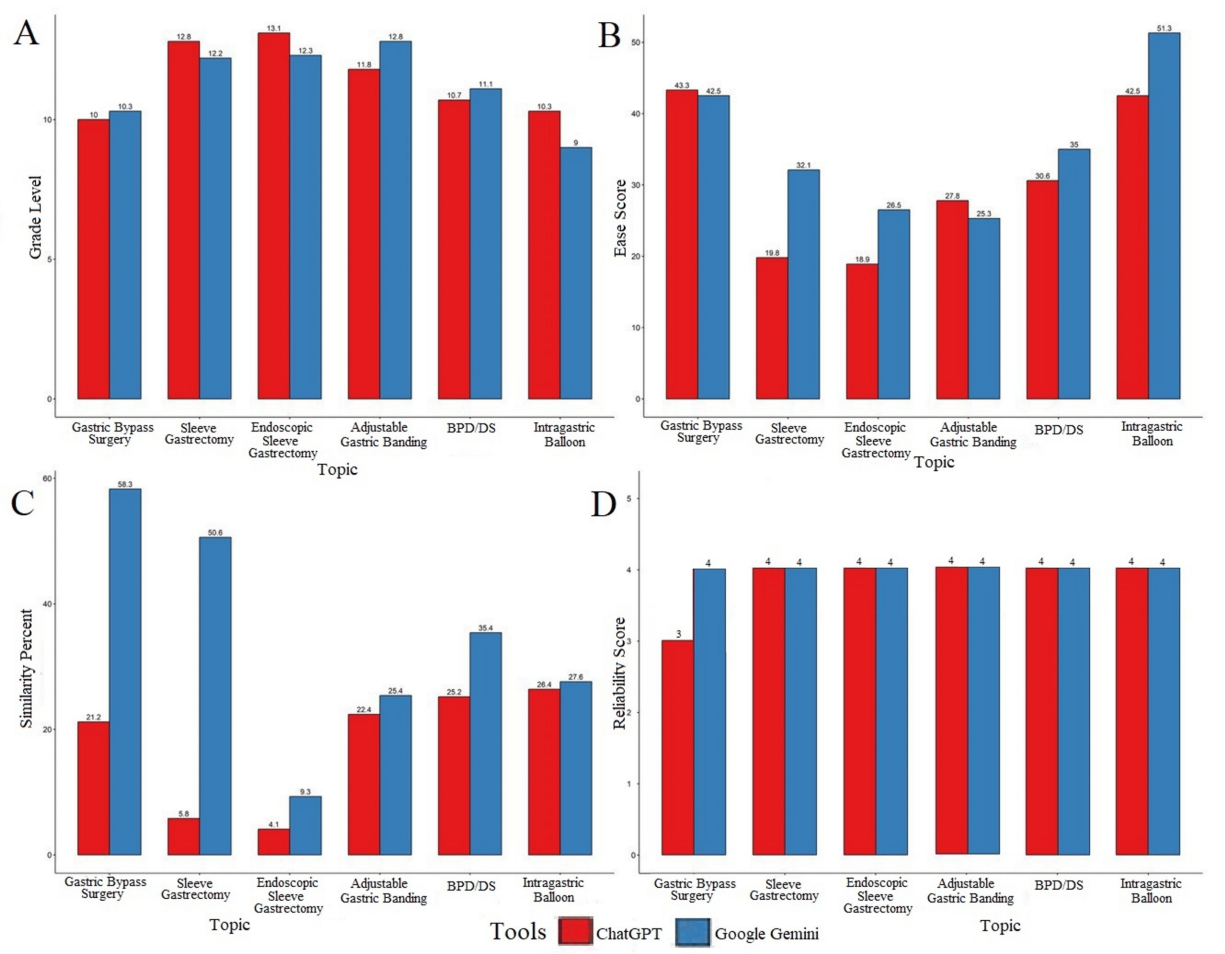
Graphical representation of comparison between grade level, ease score, similarity percent and reliability score, for the patient education guides generated by ChatGPT and Google Gemini. This figure contrasts performance metrics of ChatGPT and Google Gemini for specific medical topics, showing a graphical representation of comparison between grade level, ease score, similarity percent and reliability score for the patient education guides generated by them. It contains four bar plots, each comparing data across six medical topics: Gastric Bypass Surgery, Sleeve Gastrectomy, Endoscopic Sleeve Gastroplasty, Adjustable Gastric Banding (Lap Band), Biliopancreatic Diversion with Duodenal Switch (BPD/DS), and Intragastric Balloon. Each bar represents values from two sources: ChatGPT (red bars) and Google Gemini (blue bars). BPS/DS, Biliopancreatic diversion with duodenal switch

Figure 1A-1D is a graphical representation of the Flesch Kincaid grade level and ease score, similarity percent and mDISCERN reliability score of both the tools for the six topics considered in this study. They show similar trends in grade level across all topics indicating generally equivalent levels of complexity (Figure 1A). Google Gemini is leading in ease scores as well as similarity percent denoting ease of comprehension but possible risk of plagiarism (Figure 1B-1C). Upon scrutinizing the reliability of the instructional manuals generated, it is interesting to note almost identical scores with the exception of a lower score for ChatGPT in a singular topic (Figure 1D).

Graphical trends

Patterns of readability grade level, ease scores, similarity percentages, and mDISCERN reliability scores across all six bariatric procedures are shown in Figure 1. These graphs demonstrate comparable readability between the tools, slightly higher ease scores and similarity percentages for Gemini, and nearly identical reliability across both tools.

## Discussion

This cross-sectional study compared the patient education brochures generated by ChatGPT and Google Gemini for six bariatric procedures. The main finding is that although the two AI tools differed significantly in word count, sentence count, and sentence length, they produced broadly similar results in key domains such as ease score, reliability, grade level, and similarity percentage. These findings indicate that both AI models are capable of generating patient information material of comparable quality.

AI continues to gain prominence as a source of quick, accessible medical information [5]. Patients frequently seek information online before consulting a clinician, making the readability and reliability of such material increasingly important. Because clinicians often face challenges in simplifying complex content due to information asymmetry, AI tools may help bridge this gap by summarizing medical concepts and providing clearer explanations for patients [22]. Many healthcare organizations have recognized the need for accessible patient information and have emphasized simplicity and clarity in educational materials [23]. Our study adds to this growing area of inquiry by examining the readability and reliability of AI-generated patient brochures.

Shorter, concise text generally enhances comprehension, which is why structural metrics were compared. While the two models differed significantly in volume and sentence structure, both produced brochures with similar ease scores. However, these scores correspond to a college-level reading difficulty, indicating that individuals without higher education may find the material challenging. This highlights an important limitation of current AI-generated text for patient-facing use.

Similarity percentage, used as a proxy for textual overlap, was higher in Gemini than in ChatGPT. Since AI models are trained on pre-existing literature, some overlap is expected. However, excessive similarity carries risks in scientific communication, where originality and accuracy are essential. In this study, Gemini showed greater overlap, whereas ChatGPT generated more original phrasing.

The modified DISCERN score further supported the reliability of both tools. Scores of 3.83 and 4 suggest that the brochures contained information of good quality, relevant and useful to patients [21]. These findings align with prior work, such as that by Adithya S et al. (2024), which reported similar results for AI-generated educational content [24]. Although the study used two widely known AI tools, the findings should be interpreted cautiously. The analysis focused only on two platforms, one specialty domain, and English-language content; therefore, broad generalizability cannot be assumed [25]. Different conditions, languages, or user prompts may yield different outcomes. Additionally, an older version of ChatGPT was evaluated, which may not reflect current model capabilities.

Recent literature evaluating multiple large language model (LLM) chatbots further supports the need to assess readability alongside reliability when using AI for patient education. Mondal et al. [26] assessed the ability of several LLM chatbots to generate plain language summaries and demonstrated notable variability in readability across models, with Claude consistently achieving lower Flesch-Kincaid grade levels compared to other chatbots, indicating superior accessibility for lay audiences. Similarly, Sarangi et al. [27], in a radiology-focused evaluation of LLM-generated plain language summaries, reported that Claude outperformed other models in producing content with simpler language and improved readability while maintaining informational accuracy. These findings suggest that inter-model differences significantly influence the suitability of AI-generated patient education materials and that selection of the AI tool itself plays a critical role in achieving appropriate health literacy standards. In contrast, the present study evaluated only ChatGPT and Google Gemini, both of which produced content at a higher-than-recommended reading level, underscoring the need for broader multi-model comparisons in future research.

The study has several limitations: the use of only two AI tools, evaluation of one clinical specialty, reliance on English text, and possible differences in translation or colloquial expression that were not captured. Future research should expand to multiple specialties, additional AI platforms, and multilingual analysis to better understand the diverse clinical and patient contexts in which AI-generated materials may be used.

Despite these limitations, the study highlights important future directions. AI-generated materials show promise but should serve as a complement, not a replacement, to clinician-led patient education. Improving readability and ensuring human oversight will be essential to make AI-generated brochures more accessible, accurate, and safe for widespread patient use.

## Conclusions

Our study demonstrates that both ChatGPT and Google Gemini can generate reliable and reasonably comprehensible patient education brochures for bariatric surgery. While some statistical differences were noted in text length and sentence structure, overall readability, reliability, and quality were comparable and rated as good. This suggests that AI tools can serve as valuable adjuncts in improving patient awareness and understanding of complex surgical procedures. However, the current readability remains at a college level, which may pose challenges for patients with lower literacy levels.

Further research should focus on expanding the scope to include additional AI platforms, diverse medical conditions, and multilingual adaptations to better reflect real-world patient populations. Incorporating physician oversight and tailoring readability to different literacy levels will be essential for safe and effective implementation. While AI cannot replace the empathy and context provided by clinicians, it has strong potential to complement traditional patient education, bridge knowledge gaps, and enhance shared decision-making in clinical care.
